# Inhibition of oxidative metabolism by nitric oxide restricts EMCV replication selectively in pancreatic beta-cells

**DOI:** 10.1074/jbc.RA120.015893

**Published:** 2021-01-13

**Authors:** Joshua D. Stafford, Chay Teng Yeo, John A. Corbett

**Affiliations:** Department of Biochemistry, Medical College of Wisconsin, Milwaukee, Wisconsin, USA

**Keywords:** β-cells, nitric oxide, EMCV, mitochondrial metabolism, innate immunity, autoimmune diabetes, diabetes, glucose metabolism, plus-stranded RNA virus, mitochondria, beta cell, virus replication

## Abstract

Environmental factors, such as viral infection, are proposed to play a role in the initiation of autoimmune diabetes. In response to encephalomyocarditis virus (EMCV) infection, resident islet macrophages release the pro-inflammatory cytokine IL-1β, to levels that are sufficient to stimulate inducible nitric oxide synthase (iNOS) expression and production of micromolar levels of the free radical nitric oxide in neighboring β-cells. We have recently shown that nitric oxide inhibits EMCV replication and EMCV-mediated β-cell lysis and that this protection is associated with an inhibition of mitochondrial oxidative metabolism. Here we show that the protective actions of nitric oxide against EMCV infection are selective for β-cells and associated with the metabolic coupling of glycolysis and mitochondrial oxidation that is necessary for insulin secretion. Inhibitors of mitochondrial respiration attenuate EMCV replication in β-cells, and this inhibition is associated with a decrease in ATP levels. In mouse embryonic fibroblasts (MEFs), inhibition of mitochondrial metabolism does not modify EMCV replication or decrease ATP levels. Like most cell types, MEFs have the capacity to uncouple the glycolytic utilization of glucose from mitochondrial respiration, allowing for the maintenance of ATP levels under conditions of impaired mitochondrial respiration. It is only when MEFs are forced to use mitochondrial oxidative metabolism for ATP generation that mitochondrial inhibitors attenuate viral replication. In a β-cell selective manner, these findings indicate that nitric oxide targets the same metabolic pathways necessary for glucose stimulated insulin secretion for protection from viral lysis.

Autoimmune diabetes is characterized by the selective destruction of insulin producing β-cells that occurs during an inflammatory reaction in and around pancreatic islets. Although genetic factors contribute to disease development (1, 2), the lower than expected incidence of diabetes development between monozygotic twins supports a role for environmental factors as potential precipitating events that trigger autoimmunity against β-cells (3). Viral infection is one environmental factor that has been proposed to contribute to diabetes development. Enteroviruses are nonenveloped, positive-sense, single-stranded RNA viruses of the Picornavirus family that include coxsackie (CBV)-, polio-, and human rhinoviruses. Members of this virus family have received considerable attention as potential environmental factors that trigger type 1 diabetes (4, 5). Enterovirus tropism for islets and β-cells has been observed in pancreatic sections of neonates who perished following a lethal infection (6, 7). Encephalomyocarditis virus (EMCV) is a member of the Picornavirus family that is capable of infecting, replicating in, and lysing mouse β-cells, and in genetically susceptible strains of mice (DBA/2J, SJL/J and SWR/J) it will induce diabetes (8, 9).

Proinflammatory cytokines are primary mediators of inflammation and they have been shown to cause damage to pancreatic β-cells. Treatment of rodent or human islets with a combination of IL-1, TNFα, and IFN-γ results in an inhibition of insulin secretion, oxidative metabolism, protein synthesis, and the induction of DNA damage. This damage is the result of β-cell expression of iNOS and production of micromolar levels of nitric oxide (10, 11, 12, 13, 14, 15). Although nitric oxide mediates the inhibitory actions of cytokines on β-cell function, these actions are reversible (16, 17). Nitric oxide also activates several prosurvival responses including DNA repair pathways and a protective unfolded protein response (UPR) (18, 19, 20, 21) as well as the inhibition of pathways leading to cell death including caspase activation (22, 23, 24). It is only after β-cells no longer produce nitric oxide, which occurs following prolonged exposures to cytokines of 36 h or longer (14), that the actions of cytokines become irreversible and β-cells are commitment to death (14, 17).

In reevaluating these contrasting responses, we reasoned that cytokine signaling in β-cells could play a physiologically protective role in promoting β-cell survival, and in support of this hypothesis, we have recently shown that nitric oxide attenuates EMCV replication in insulinoma MIN6 cells and mouse islets by inhibiting mitochondrial oxidative metabolism (25). Inhibitors of the electron transport chain and mitochondrial uncouplers attenuate EMCV replication and EMCV-mediated MIN6 cell lysis in a manner like nitric oxide. The purpose of the current study was to determine if the actions of nitric oxide are cell type selective, as the regulation of oxidative metabolism in β-cells differs from most other cell types. We show that the inhibitory actions of nitric oxide are selective for β-cells and are associated with the coupling of glycolysis and mitochondrial oxidative metabolism that is essential for glucose-dependent insulin secretion by β-cells (26, 27). In pancreatic β-cells, 90% of the carbons of glucose are oxidized to CO_2_, and this occurs in a concentration-dependent manner. Intermediary metabolism in non-β-cells is flexible in that glycolysis and mitochondrial oxidative metabolism are uncoupled, allowing most non-β-cell types to increase glycolytic flux under conditions in which mitochondrial oxidative metabolism is impaired (27, 28). In this report, we show that when non-β-cells are unable to maintain this metabolic flexibility, nitric oxide and inhibitors of mitochondrial oxidative metabolism attenuate EMCV replication and prevent EMCV-induced cell lysis in a manner similar to what we observe in β-cells. These findings indicate that the inhibitory actions of nitric oxide on EMCV replication are selective for pancreatic β-cells and the mechanism of inhibition is associated with the metabolic coupling of intermediary metabolism that is unique to β-cells and necessary for proper glucose-stimulated insulin secretion

## Results

### Cell type specificity in the inhibition of EMCV replication by nitric oxide

**Figure 1:**
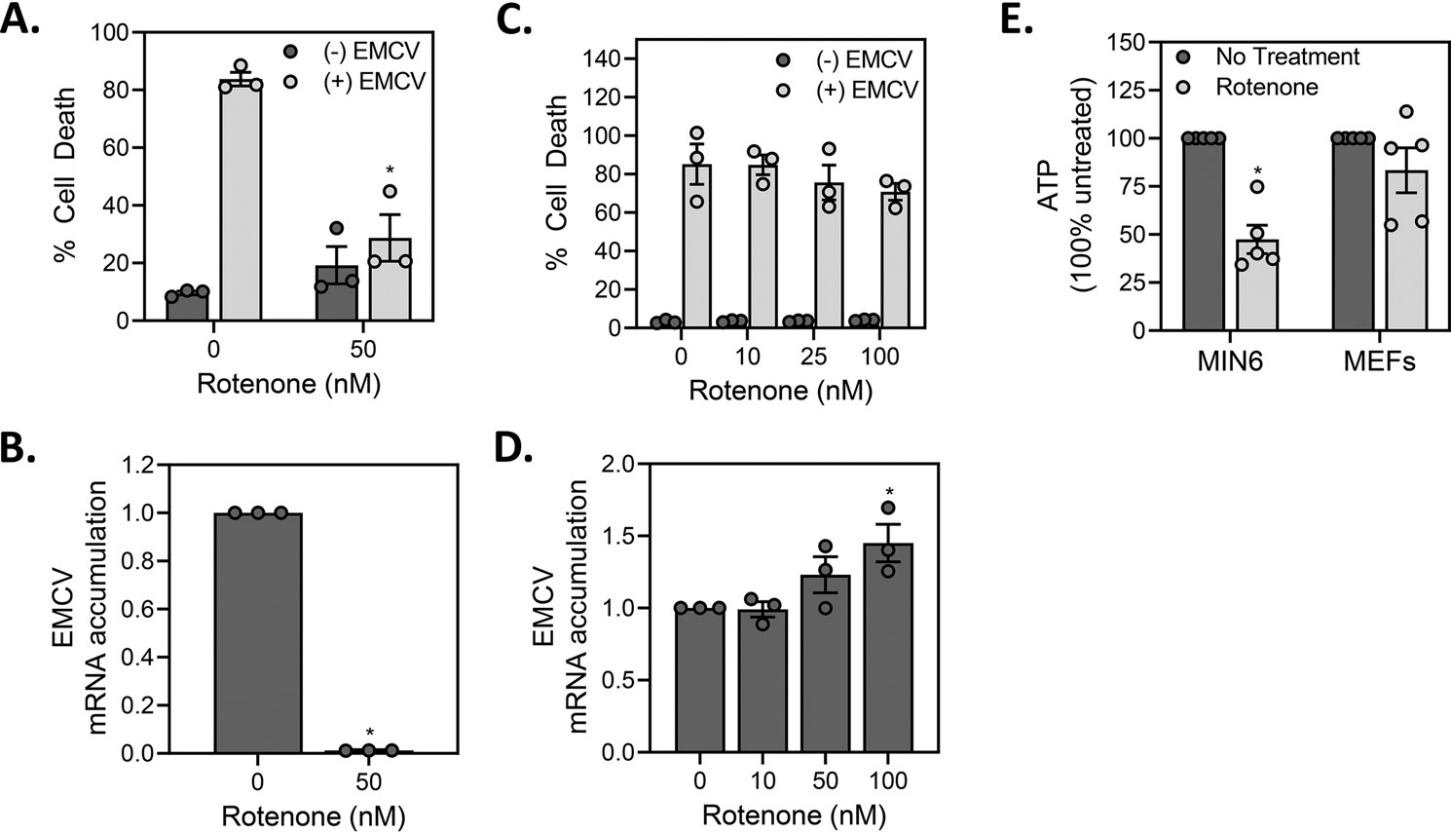
β**-cell-selective inhibition of EMCV replication by inhibitors of mitochondrial respiration.***A*, MIN6 cells (50,000/100 μl medium) were infected with 5 MOI EMCV in the presence or absence of 50 nm rotenone and cell death was measured by SYTOX fluorescence 18 h post-infection. *B*, MIN6 cells (200,000/400 μl medium) were infected with 5 MOI EMCV and EMCV mRNA (VP1) accumulation was determined by qRT-PCR 12 h post-infection. *C*, MEF (10,000/100 μl medium) were infected with 0.1 MOI EMCV in the presence of increasing concentrations of rotenone and cell death was measured by SYTOX fluorescence 24 h post-infection. *D*, MEF (40,000/400 μl medium) were infected with 0.1 MOI EMCV in the presence of increasing concentrations of rotenone and EMCV mRNA accumulation was determined by qRT-PCR 12 h post-infection. *E*, MIN6 cells (1.0 × 10^6^/2 ml medium) and MEF (200,000/2 ml medium) were treated with 100 nm rotenone for 2 h and cellular levels of ATP were determined by HPLC analysis and normalized to total protein concentration. Results are the average ± S.E. of 3–5 independent experiments, statistically significant differences are indicated (*, p < 0.05).

We have shown that nitric oxide attenuates EMCV replication and EMCV-mediated lysis of pancreatic β-cells by inhibiting mitochondrial oxidative metabolism (25). Because the regulation of intermediary metabolism in β-cells differs from most other cell types, we examine whether the inhibition of mitochondrial respiration is a general antiviral response observed in all cell types or selective for β-cells. Consistent with our previous studies (25), rotenone, an inhibitor of complex I of the electron transport chain, attenuates EMCV-mediated MIN6 cell lysis and EMCV mRNA accumulation (Fig. 1*A* and *B*) and this action appears to be selective for β-cells as rotenone does not modify EMCV replication or the lysis of MEF infected with EMCV (Fig. 1*C* and *D*).

An essential attribute of anaerobic metabolism that is observed in most cell types is enhanced glycolysis under conditions of impaired mitochondrial oxidative metabolism (28). β-cells lack this metabolic flexibility, as the coupling of glycolytic and mitochondrial oxidation of glucose is an essential regulatory feature of glucose-induced insulin secretion (26). A consequence of this lack of flexibility is the decrease in ATP levels in β-cells experiencing impaired mitochondrial oxidative capacity in response to nitric oxide (27, 29) or inhibitors of mitochondrial respiration such as rotenone (Fig. 1*E*). In cells that maintain metabolic flexibility such as MEFs, the inhibition of mitochondrial respiration does not modify ATP levels (Fig. 1*E*), consistent with our previous observations (27). These data correlate the β-cell selective inhibition of EMCV replication by mitochondrial respiratory chain inhibitors with decreases in ATP levels that are because of an inability of β-cells to compensate for the inhibition of mitochondrial oxidative metabolism with increases in glycolytic flux (27).

### Inhibition of EMCV replication in galactose-cultured MEFs

**Figure 2:**
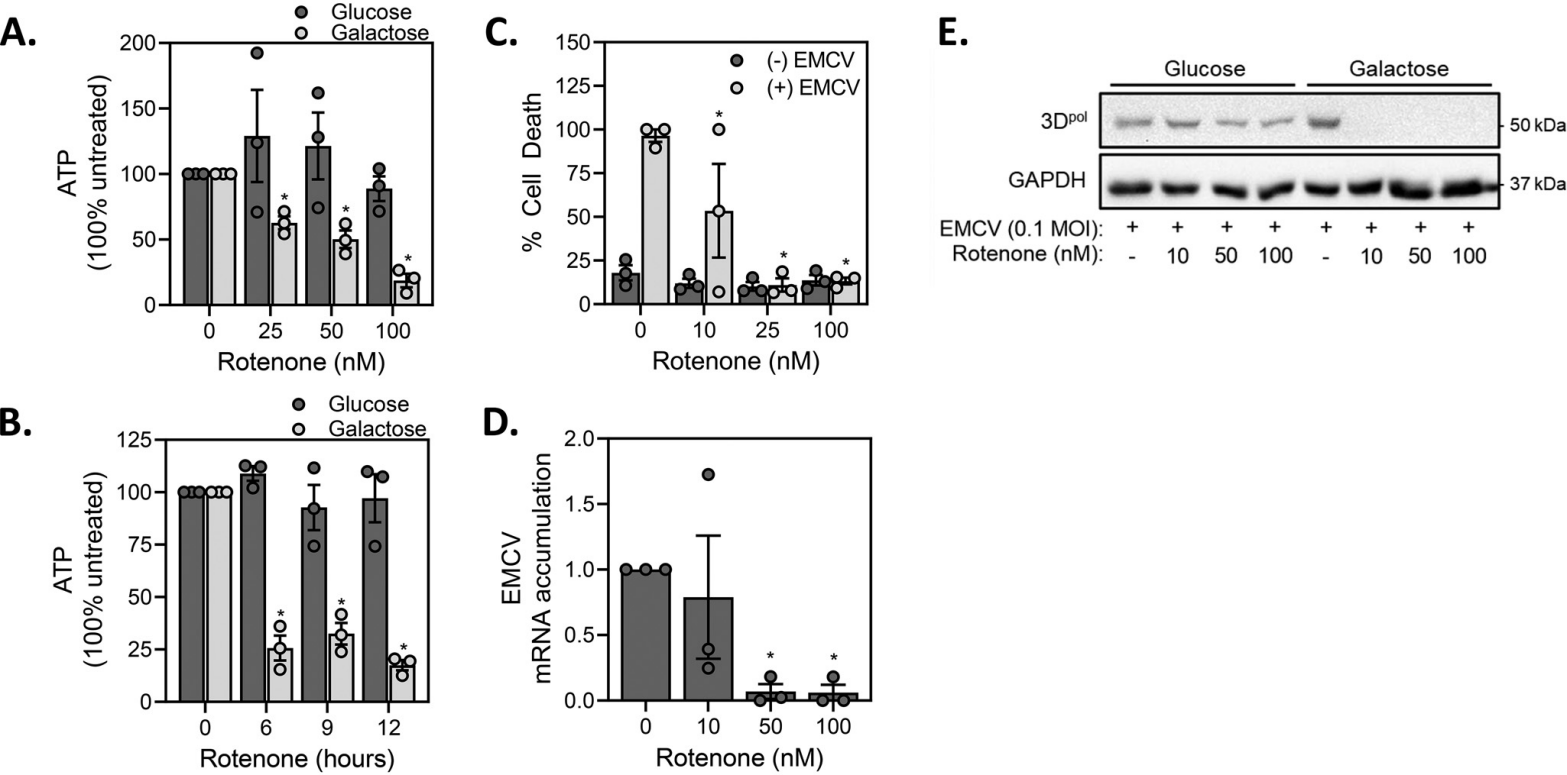
**Rotenone inhibits EMCV replication in galactose but not glucose cultured MEF.***A*, *B*, MEF were cultured in either glucose- or galactose-containing media (200,000/2 ml medium) with or without the indicated concentrations of rotenone for 9 h (*A*) or with 100 nm rotenone for the indicated times (*B*) and then cellular levels of ATP were determined by HPLC analysis and normalized to total protein concentration. *C*, MEF cultured in glucose-free, galactose-containing media (10 mm) (10,000/100 μl medium) were infected with 0.1 MOI EMCV in the presence of increasing concentrations of rotenone and cell death was determined by SYTOX fluorescence 24 h post-infection. *D*, *E*, MEF cultured in galactose-containing medium (40,000/400 μl medium) were infected with 0.1 MOI EMCV in the presence of increasing concentrations of rotenone and EMCV mRNA accumulation was determined by qRT-PCR 12 h post-infection (*D*) and the accumulation of viral polymerase (3D^pol^) was determined by Western blotting analysis 18 h post-infection (*E*). Results are the average ± S.E. of 3 independent experiments (*A*-*D*) or representative (*E*) of 3 independent experiments. Statistically significant differences are indicated (*, p < 0.05).

When cultured in glucose, most cell types produce ATP via glycolysis (30); however, this shifts to mitochondrial oxidative metabolism when cells are cultured with galactose as the primary carbon source (28). This occurs because mitochondrial oxidation of glutamine becomes more favorable than glycolytic use of this alternative carbon source (28, 31, 32). Glutamine is deaminated to glutamate by glutaminase and then imported into the mitochondria where it enters the tricarboxylic acid (TCA) cycle to generate reducing equivalents for oxidative phosphorylation (28, 31, 32). This shifts ATP generation from glycolysis to mitochondrial oxidative metabolism and prevents glycolytic compensation for impaired mitochondrial respiration (27, 28, 31, 32). In both a time- and concentration-dependent manner rotenone decreases ATP levels and attenuates the lysis of EMCV-infected MEF when cultured in galactose-, but not when cultured in glucose-containing medium (Fig. 2*A*–*B*). The shift in metabolic burden to the mitochondria in galactose-cultured MEFs correlates with an inhibition of cell lysis by rotenone following EMCV infection (Fig. 2*C*). Rotenone also attenuates EMCV mRNA accumulation and the expression of the viral polymerase protein (3D^pol^) in galactose-cultured but not in glucose-cultured MEFs (Fig. 2*D* and *E*). These data indicate that shifting the metabolic burden to mitochondrial ATP generation in non-β-cells renders these cells sensitive to rotenone as an inhibitor of EMCV replication and EMCV-mediated lysis in a manner like β-cells.

**Figure 3:**
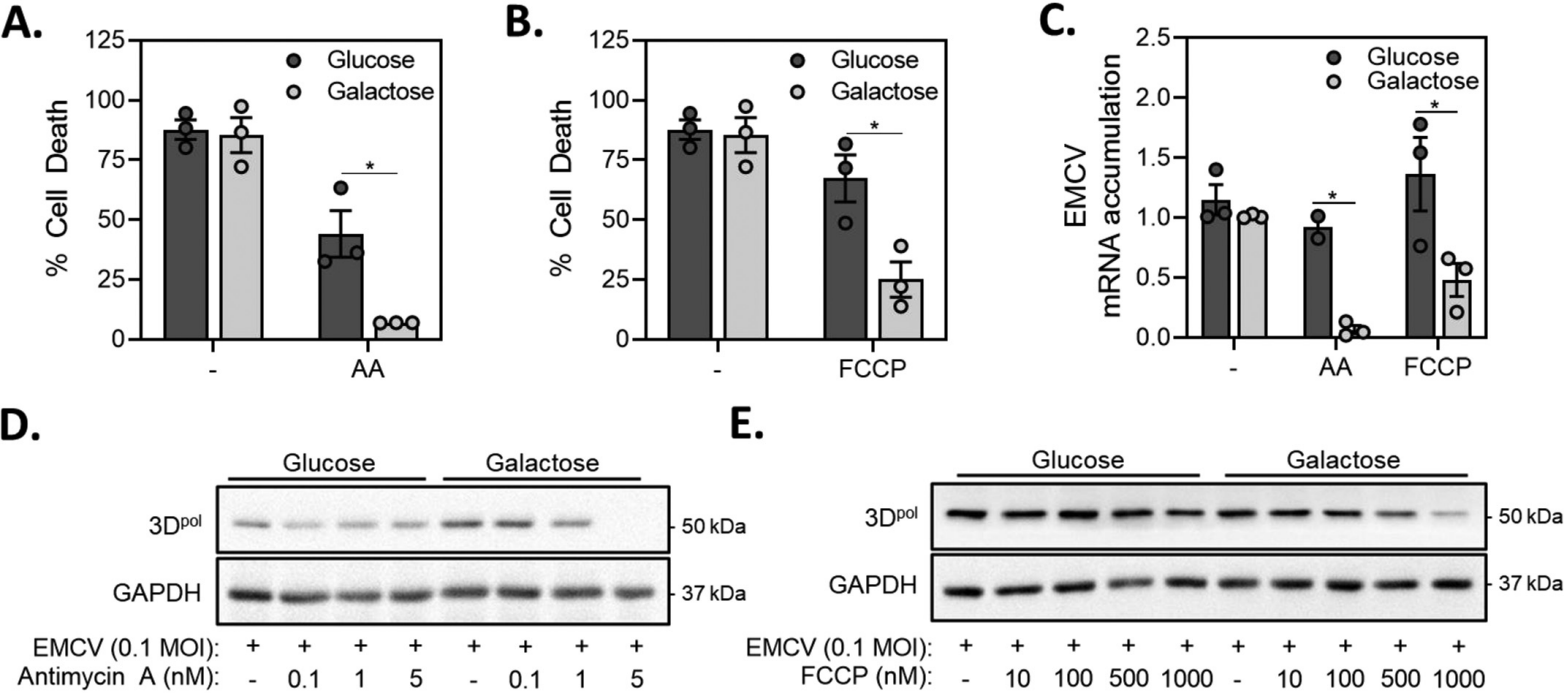
**Mitochondrial respiratory inhibitors attenuate virus replication in galactose-cultured MEF.** A–E, MEF (10,000/100 μl medium) cultured in either glucose- or galactose-containing media were infected with 0.1 MOI EMCV in the presence of 5 nm antimycin A (*A*) or 1 μm FCCP (*B*) and cell death was measured by SYTOX fluorescence 24 h post-infection. The accumulation of EMCV mRNA was determined by qRT-PCR 12 h post-infection (*C*) and viral protein accumulation (3D^pol^) was determined by Western blotting 18 h post-infection (*D*, *E*). Results are the average ± S.E. of 3 independent experiments (*A*–*C*) or representative (*D*–*E*) of 3 independent experiments. Statistically significant differences are indicated (*, p < 0.05).

Much like the actions of rotenone and nitric oxide, disruption of electron transport at complex III with antimycin A and the uncoupling agent FCCP provide greater protection against EMCV-mediated lysis in galactose-cultured, as compared with glucose-cultured MEFs (Fig. 3*A* and *B*). Antimycin A and FCCP also attenuate EMCV mRNA accumulation (Fig. 3*C*) and 3D^pol^ expression in galactose-cultured MEFs (Fig. 3*D*–*E*). These findings are consistent with the inhibitory effects of antimycin A and FCCP on EMCV replication in β-cells (25) and associate decreased levels of ATP with an inhibition of EMCV replication and cell lysis.

### Role of AMPK activation in the protection of β-cells from EMCV

**Figure 4:**
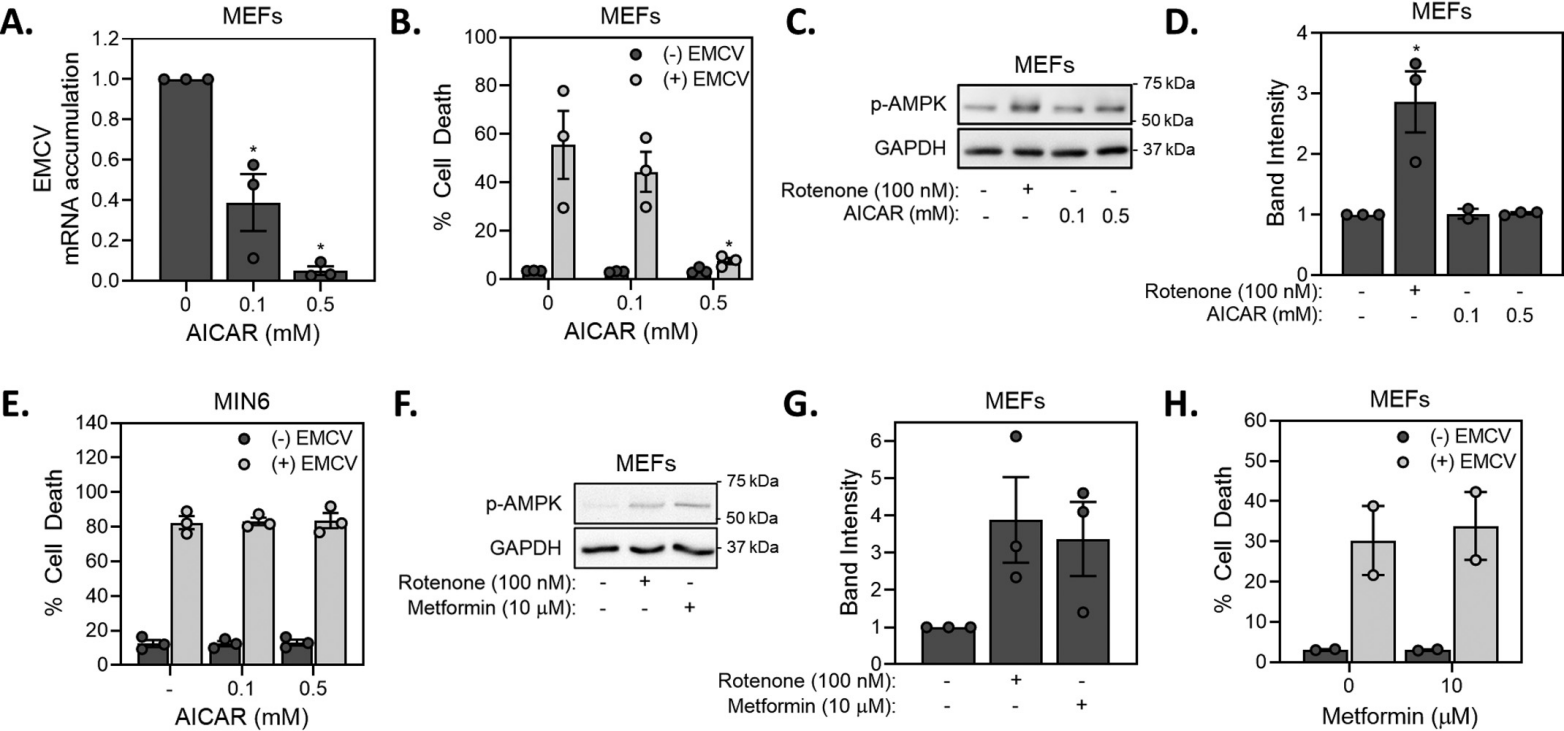
**Role of AMPK in the inhibition of EMCV replication.***A*, MEF (40,000/400 μl medium) were infected with 0.1 MOI EMCV in the presence of increasing concentrations of AICAR and EMCV mRNA accumulation was determined by qRT-PCR 12 h post-infection. *B*, MEF (10,000/100 μl medium) were infected with 0.1 MOI EMCV in the presence of increasing concentrations of AICAR and cell death was determined by SYTOX fluorescence 24 h post-infection. (*C*, *D*, *F*, *G*) MEF (40,000/400 μl medium) were treated with AICAR (*C*, *D*) or metformin (*F*, *G*) for 2 h and phosphorylation of AMPK was determined by Western blotting and quantified using densitometry. The effects of rotenone are shown as a positive control. *E*, MIN6 cells (50,000/100 μl medium) were infected with 5 MOI EMCV in the presence of increasing concentrations of AICAR and cell death was determined by SYTOX fluorescence 18 h post-infection. *H*, MEF (10,000/100 μl medium) were infected with 0.1 MOI EMCV in the presence or absence of metformin and cell death was determined by SYTOX fluorescence 24 h post-infection. Results are the average ± S.E. of 2–3 independent experiments (*A*, *B*, *D*, *E*, *G*, *H*) or representative (*C*, *F*) of 3 independent experiments. Statistically significant differences are indicated (*, p < 0.05).

One potential target of decreased levels of ATP is AMP-activated protein kinase (AMPK). This cellular energy sensor is activated by an increase in the AMP/ATP ratio and once activated it attenuates energy-consuming processes, such as protein and fatty acid synthesis, and enhances energy preserving pathways such as autophagy and glycolysis (33). Activators of AMPK have been shown to protect cells during coxsackievirus infection (a picornavirus similar to EMCV) by attenuating fatty acid synthesis (34, 35) and in a concentration-related manner, the AMPK activator 5-aminoimidazole-4-carboxamide ribonucleotide (AICAR) attenuates EMCV replication in MEFs (Fig. 4*A*). Consistent with the inhibition of replication, AICAR also attenuates EMCV-mediated MEF lysis (Fig. 4*B*). Unfortunately, concentrations of AICAR that attenuate MEF lysis and EMCV replication, fail to activate AMPK as determined by phosphorylation (Fig. 4*C*–*D*). Further, in glucose-containing medium, rotenone fails to protect MEFs from EMCV-mediated lysis (Fig. 1*C*), even though it stimulates AMPK phosphorylation (Fig. 4*C* and *D*). These findings suggest that AICAR has off-target AMPK-independent effects on EMCV replication. Consistent with this view, AICAR fails to protect MIN6 cells from EMCV-mediated lysis (Fig. 4*E*) and metformin, a second activator of AMPK (and also an inhibitor of complex I) which stimulates AMPK phosphorylation, does not prevent EMCV-mediated MEF lysis (Fig. 4*F*–*H*). These findings dissociate AMPK activation from the protective effects of AICAR on EMCV-mediated lysis in MEFs and suggest that AMPK activation itself is not sufficient to provide protection from EMCV.

### Nitric oxide protects galactose-cultured MEFs from EMCV-mediated cell lysis

**Figure 5:**
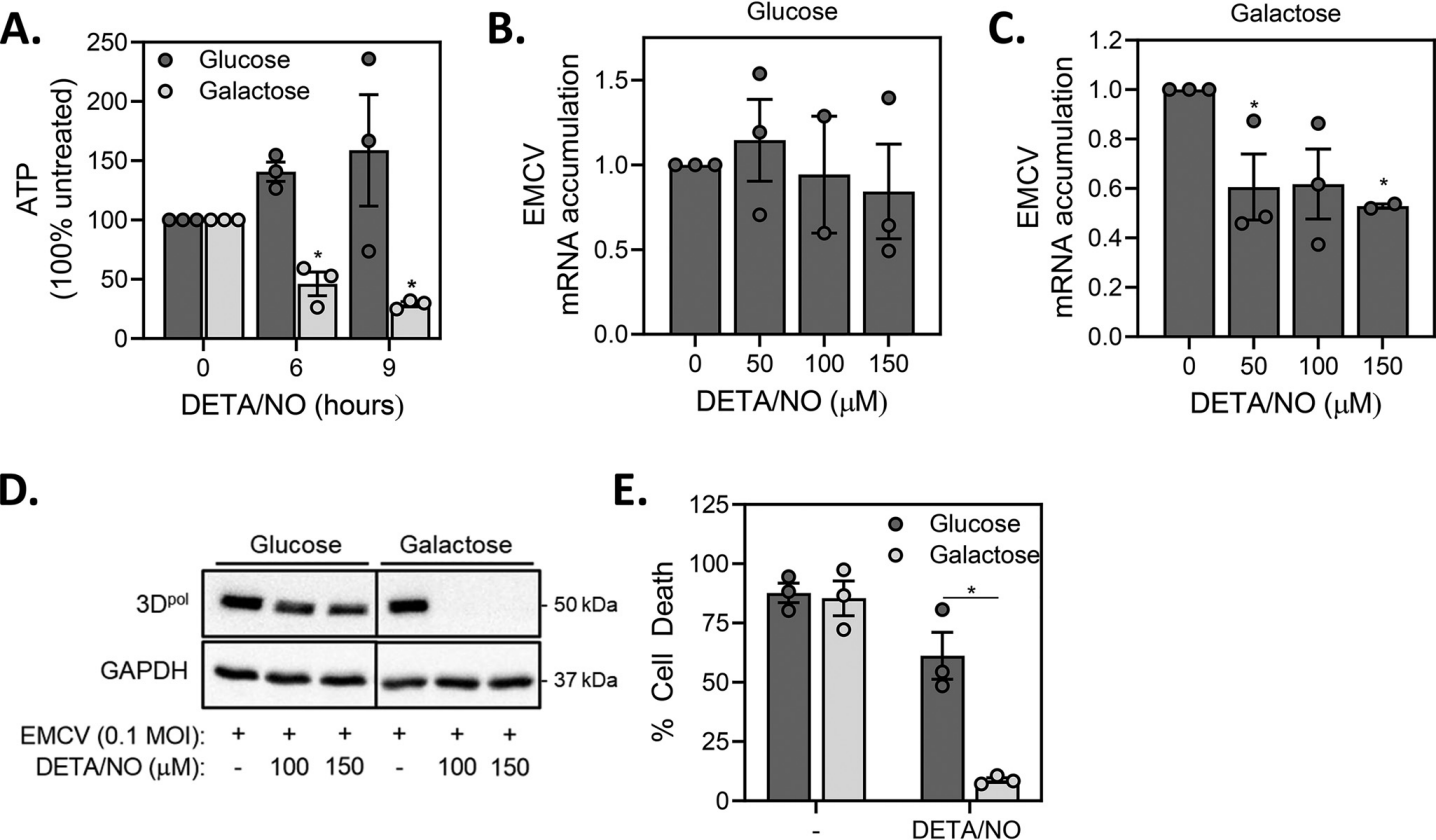
**Nitric oxide attenuates EMCV replication in galactose-cultured MEF.***A*, MEF, cultured in either glucose- or galactose-containing media (200,000/2 ml medium) were treated with 150 μm DETA/NO for the indicated times, and then cellular ATP levels were determined by HPLC analysis and normalized to total protein concentrations. *B*–*D*, MEF (40,000/400 μl medium) cultured in glucose- or galactose-containing media were infected with 0.1 MOI EMCV in the presence of increasing concentrations of DETA/NO and EMCV mRNA accumulation was determined by qRT-PCR 12 h post-infection (*B*, *C*) and EMCV protein (3D^pol^) accumulation was determined by Western blotting analysis 18 h post-infection (*D*). *E*, MEF cells cultured in either glucose- or galactose-containing media (10,000/100 μl medium) were infected with 0.1 MOI EMCV in the presence of 150 μM DETA/NO and cell death was determined by SYTOX fluorescence 24 h post-infection. Results are the average ± S.E. of 3 independent experiments (*A–C*, *E*) or representative (*D*) of 3 independent experiments. Statistically significant differences are indicated (*, p < 0.05).

Nitric oxide, at iNOS-derived levels, attenuates EMCV replication and β-cell lysis (25), but does not modify EMCV replication or the lysis of MEFs when cultured in glucose-containing media (Fig. 5). In MEFs forced to generate ATP via mitochondrial respiration (culturing in galactose, Fig. 5*A*), nitric oxide decreases ATP levels in a time-dependent manner that is similar to the effects of rotenone shown in Fig. 2. The decrease in ATP is associated with an inhibition of EMCV mRNA accumulation (Fig. 5*B*–*C*) and the expression of EMCV 3D^pol^ (Fig. 5*D*) and the inhibition of EMCV-mediated MEF cell lysis (Fig. 5*E*). These findings suggest that the β-cell selective inhibition of EMCV replication by nitric oxide and other inhibitors of mitochondrial oxidative metabolism is associated the metabolic coupling of glucose metabolism that occurs in these insulin-producing cells. In support of this hypothesis, EMCV replication in non-β-cells is attenuated by inhibitors of mitochondrial metabolism and nitric oxide when forced to derive ATP via mitochondrial oxidation.

### Nitric oxide and rotenone inhibit EMCV replication selectively in mouse β-cells

**Figure 6:**
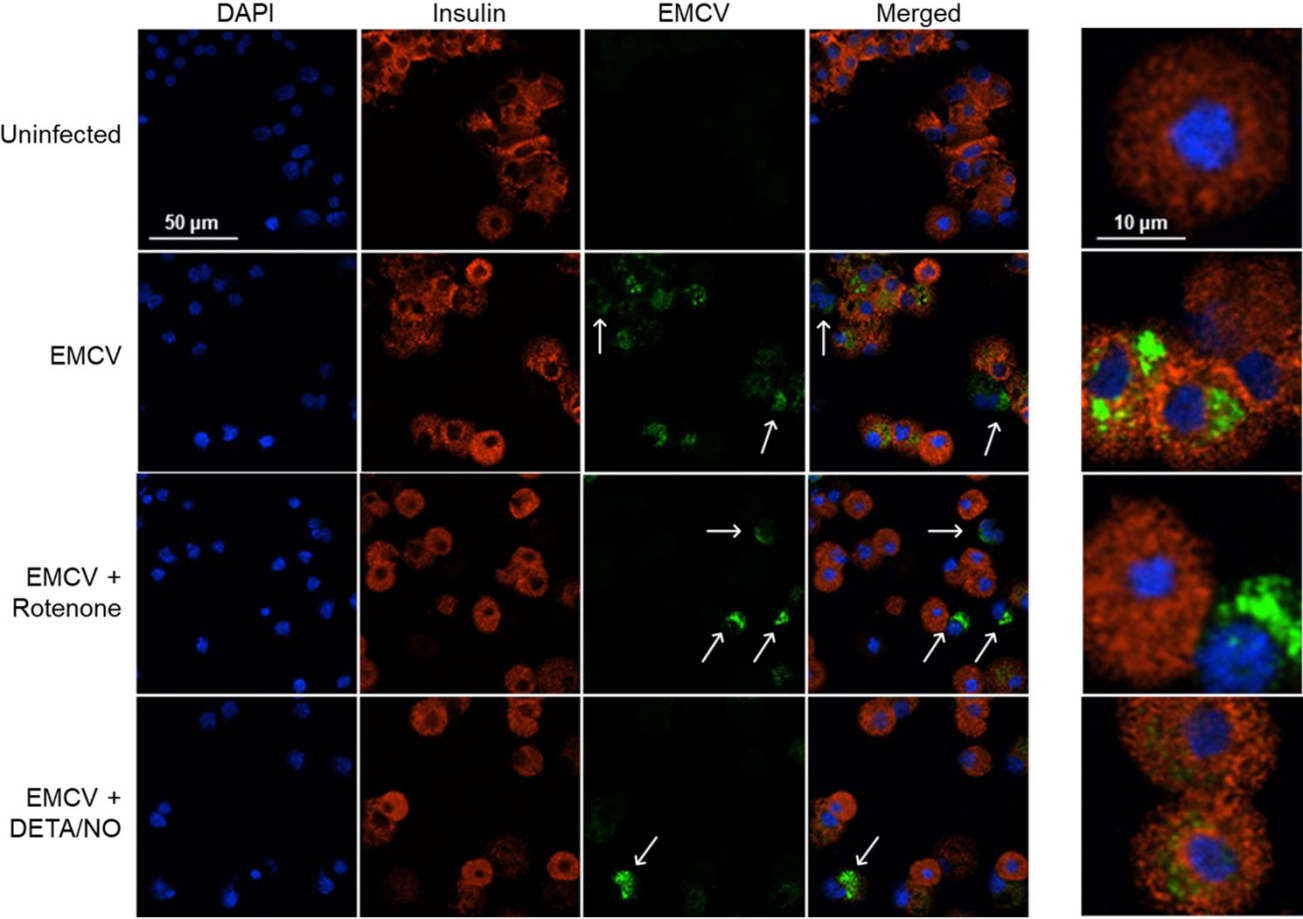
**Inhibition of mitochondrial oxidative metabolism attenuates EMCV replication selectively in** β**-cells.** Dispersed mouse islet cells were infected with 5 MOI EMCV for 15 h in the presence or absence of 50 nm rotenone or 150 μm DETA/NO. Cell nuclei were identified using DAPI fluorescence (*blue*), cells containing EMCV were identified by immunostaining for EMCV capsid protein (*green*), and β-cells were identified by immunostaining for insulin (*red*). Cells were visualized using a Nikon Eclipse 90i confocal microscope (100× with 2× field zoom). Results are representative of three independent experiments.

Islets represent a heterogenous population of endocrine and nonendocrine cell types, of which β-cells comprise 60–80% of these cells (36). This is the ideal experimental system to directly examine the cell type specific effects of inhibiting mitochondrial respiration on EMCV replication. To examine this question, mouse islets were dispersed into individual cells and then infected with EMCV in the presence or absence of rotenone or DETA/NO. Fifteen hours post-infection, immunofluorescence spectroscopy was used to identify cells expressing EMCV capsid protein (AF-488-conjugated secondary antibody, green) and cells expressing insulin (CY3-conjugated secondary antibody, red). Insulin-containing β-cells (red) are the most abundant islet cell type to be infected with EMCV; however, EMCV capsid protein is also expressed in cells that do not contain insulin (arrows, Fig. 6). Consistent with β-cell selective inhibition of EMCV replication by inhibitors of mitochondrial oxidative metabolism, rotenone and DETA/NO attenuate EMCV capsid protein fluorescence in insulin-containing cells, without modifying the fluorescence in non-insulin-containing cells (arrows, Fig. 6). These findings provide further support that the inhibition of mitochondrial oxidative metabolism impairs EMCV replication selectively in β-cells without modifying EMCV replication in non-β-cells of the islet.

## Discussion

The physiological role of pancreatic β-cells is to maintain whole body glucose homeostasis by sensing blood glucose levels and releasing the precise amount of insulin needed to stimulate glucose disposal in the periphery. This process is controlled by multiple mechanisms. The first is glucose sensing, where the high *K_m_* of the GLUT2 transporter (11-15 mm) and of glucokinase (5.5 mm) control glucose uptake and phosphorylation in a concentration-dependent manner (26). The rates of glucose oxidation determine the amount of insulin that is released. In β-cells, 90% of the carbons of glucose are oxidized to CO_2_ upon supply of substrate (37, 38), such that the amount of insulin that is released is proportional to the rates of glucose oxidation (37, 38). In most cell types, glycolysis and mitochondrial respiration are not coupled, allowing most cells to increase glycolytic metabolism under conditions of impaired mitochondrial respiration (*e.g.* absence of oxygen). This is possible because most cell types use lactate dehydrogenase to regenerate NAD^+^, a required cofactor for GAPDH. In contrast to most cell types, β-cells express lactate dehydrogenase at very low levels, and this, in part, is responsible for the strict coupling of glycolysis and mitochondrial oxidation (37, 38, 39).

Cytokines such as IL-1 and IFN-γ are known to inhibit insulin secretion in a nitric oxide-dependent manner (11, 40, 41), as they stimulate iNOS expression and production of nitric oxide to levels sufficient to inhibit mitochondrial respiration (aconitase and complex IV of the electron transport chain) and decrease ATP (12). Based on these and many additional studies, cytokines have been viewed as potential contributors to β-cell damage during the development of autoimmune diabetes (42, 43, 44). While nitric oxide contributes to cytokine-mediated β-cell damage, we have recently identified a role for iNOS-derived nitric oxide in the antiviral defense against picornaviruses, specifically EMCV (25). In this study, we show that nitric oxide attenuates EMCV replication by inhibiting mitochondrial oxidative metabolism (25). In the current report, we extend these original observations to show that the inhibitory actions of iNOS-derived levels of nitric oxide on EMCV replication are selective for insulin-producing β-cells. The cell type selectivity is associated with the regulation of intermediary metabolism. Specifically, the coupling of glycolysis and mitochondrial oxidative metabolism that is used by β-cells to secrete the appropriate amount of insulin necessary to maintain normal glucose homeostasis. It is the inhibition of mitochondrial oxidative metabolism by nitric oxide and the lack of metabolic flexibility to compensate that protects β-cell against viral infection.

When produced at iNOS-derived levels, nitric oxide has been shown to limit the replication of a wide range of viruses (45, 46, 47, 48, 49, 50), and mice deficient in *Nos2* have reduced capacity for viral clearance and die by overwhelming viremia when infected with picornaviruses such as coxsackievirus B4 (CVB4) (49). We have identified CCR5 as a signaling receptor for EMCV that stimulates macrophage expression of iNOS (51). Infection of mice deficient in CCR5 results in an attenuation of iNOS expression that is associated with an 11-fold increase in EMCV titers (51). S-nitrosation of viral proteins is one mechanism by which nitric oxide limits viral replication (52). Upon entry the picornavirus (+)ssRNA genome is translated using host machinery (53, 54) as a polyprotein which is cleaved by the viral 3C protease (3C^pro^) in *cis*, and, once free, may cleave other polyproteins in *trans* (53, 55). This processing step is required for formation of functional viral proteins from the polyprotein precursor (53). 3C^pro^ is conserved among picornavirus family members and CVB4 replication has been shown to be inhibited by transnitrosating agents that target the active site cysteine (52). While sequence homology between the active sites of the CVB4 and the EMCV proteases coupled with the ability of nitric oxide to inhibit replication of both viruses would seem to implicate S-nitrosation of the EMCV protease as a possible mechanism for the inhibition of EMCV replication by nitric oxide, this post-translational modification does not explain the β-cell selective nature of this inhibition or the ability of inhibitors of mitochondrial respiration to attenuate EMCV replication in a β-cell selective manner (Fig. 5). Further, nitric oxide and mitochondrial respiratory inhibitors attenuate EMCV replication in non-β-cells forced to generate ATP via mitochondrial oxidation (galactose culture, Figure 1, Figure 2, Figure 3, 6). These findings suggest that S-nitrosation of viral polymerase is not a likely mechanism by which nitric oxide inhibits EMCV replication (56, 57, 58, 59).

The β-cell selective mechanism of action is associated with the lack of metabolic flexibility of β-cells to enhance glycolysis under conditions of impaired mitochondrial respiration. Under these conditions, the levels of ATP and NAD^+^ are decreased, as β-cells also lack lactate dehydrogenase impairing NAD^+^ regeneration (27). It is possible for most cell types to maintain ATP levels because they have the capacity to regenerate NAD^+^ under conditions of impaired mitochondrial respiration via lactate dehydrogenase. In these cells (whether MEFs or the non-β-cells found in islets) nitric oxide and inhibitors of mitochondrial respiration do not attenuate EMCV replication. When these non-β-cells are forced to utilize mitochondrial oxidative metabolism for ATP generation (Figure 2, Figure 3, 5), EMCV replication in these cells is attenuated by inhibitors of mitochondrial metabolism. These findings suggest that the same pathways that are used to control glucose-stimulated insulin secretion, when inhibited, function to limit viral replication in β-cells.

The selective advantage gained from this novel β-cell selective antiviral activity of nitric oxide may, in part, explain why β-cells are the primary endocrine cell source of nitric oxide in response to cytokines (60, 61). β-cells are a terminally differentiated, highly specialized cell type that has a limited proliferative capacity, and yet, as the only cell type that secretes insulin, they are essential for organismal survival (62). It is surprising to think that a cell type that is essential for organismal survival would respond to a cytokine with the production of a free radical that has been implicated in its demise. Importantly, the response of β-cells to cytokines appears to physiologically relevant and likely protective. Nitric oxide activates a number of signaling cascades that facilitate the recovery of oxidative metabolism, protein translation, insulin secretion, and the repair of damaged DNA, while at the same time nitric oxide limits cell death by inhibiting caspase activation (14, 16, 17, 18, 22, 23, 24, 63). Because IL-1 levels increase over 1000-fold during infection, and islets are highly vascularized, we believe that the response of β-cells to cytokines with the production of nitric oxide serves as a protective response. In this report, we show that nitric oxide, which is produced by β-cells in response to cytokines, limits viral replication in a β-cell selective manner. Future studies that are focused on the mechanisms by which the inhibition of mitochondrial oxidative metabolism limits viral replication and whether this pathway functions to limit additional members of the picornavirus family that have been associated with the development of autoimmune diabetes will shed even more light on the physiological relevance of β-cell production of nitric oxide.

## Materials and methods

### Materials and animals

Male DBA/2J mice were purchased from Jackson Laboratories (Bar Harbor, Maine) and housed in the MCW Biomedical Resource Center. All animal use and experimental procedures were approved by the Institutional Animal Care and Use Committees at the Medical College of Wisconsin.

MIN6 cells were obtained from the Washington University Tissue Culture Support Center (St. Louis, MO) and MEFs were obtained from ATCC (Manassas, VA). Connaught Medical Research Laboratories (CMRL) 1066 medium, fetal calf serum, horse serum, l-glutamine, sodium pyruvate, penicillin, streptomycin, and β-mercaptoethanol were purchased from Invitrogen. Dulbecco's modified Eagle's medium (DMEM) and Trypsin (0.05% in 0.53 mm EDTA) were purchased from Corning (Corning, NY). The nitric oxide donor (Z)-1-[N-(2-aminoethyl)-N-(2-ammonioethyl)amino]diazen-1-ium-1,2-diolate (DETA/NO) and 5-amino-1-β-D-ribofuranosyl-1H-imidazole-4-carboxamide (AICAR) were purchased from Cayman Chemical (Ann Arbor, MI). Rotenone, antimycin A, carbonyl cyanide 4-(trifluoromethoxy)phenylhydrazone (FCCP), and D-galactose were purchased from Sigma-Aldrich (St. Louis, MO). Metformin was purchased from Calbiochem. Primary antibodies that were used for these studies include: mouse anti-GAPDH (Invitrogen), mouse anti-Mengo 3D^pol^ (Santa-Cruz), rabbit anti-p-AMPK (Thr-172) (Cell Signaling), rabbit anti-Mengo capsid (a generous gift from Dr. Ann Palmenberg, University of Wisconsin, Madison, WI), guinea pig anti-insulin (DAKO) and secondary antibodies horseradish-peroxidase (HRP)-conjugated donkey anti-mouse, HRP-conjugated donkey anti-rabbit, Cy3-conjugated donkey anti-guinea pig, and FITC-conjugated anti-rabbit (Jackson ImmunoResearch Laboratories, West Grove, PA) antibodies.

### Islet isolation, dispersion, and cell culture

Islets from adult, male DBA/2J mice were isolated and cultured as described previously (64, 65). Prior to experimentation, islets were dispersed into single cells by incubation in 0.48 mm EDTA in PBS followed by disruption in 1 mg/ml trypsin in Ca^2+^/Mg^2+^-free Hank's balanced salt solution. MIN6 cells were maintained in DMEM containing 10% heat-inactivated fetal bovine serum, 2 mm l-glutamine, and 1 mm sodium pyruvate. MEF cells were maintained in DMEM containing 10% heat-inactivated fetal bovine serum, 1 mm pyruvate, 2 mm l-glutamine, and 10 mm HEPES. Unless otherwise indicated, all experiments including MEF cells were conducted in glucose-containing media. Both MIN6 and MEF cells were incubated at 37 °C under an atmosphere of 5% CO_2_. MIN6 cells were cultured for at least 6 h prior to the initiation of experiments while MEFs were plated 2 h prior to the initiation of experiments. MIN6 and MEF cells were removed from growth flasks by treatment with 0.05% trypsin in 0.53 mm EDTA at 37 °C for 5 min, washed twice, and plated at the indicated concentrations.

### EMCV propagation and infection

The B and D variants of EMCV were a generous gift from Dr. Ji-Won Yoon (University of Calgary, Calgary, Alta., Canada) and have been previously described (66). Cell monolayers were infected with EMCV at the indicated multiplicity of infection for 1 h at 37 °C prior to washing and replacing of media for continued culture for the indicated times. Cell lines were infected with EMCV-B and mouse islet cells were infected with EMCV-D.

### Cell death assay

Cell death was determined using the SYTOX Green nucleic acid stain (Invitrogen) as previously described (67).

### Real time PCR

Total RNA was purified from cell lysates using the RNeasy Mini Kit (Qiagen) according to manufacturer's instructions. DNase digestion was performed using Turbo DNA-free procedure (Applied Biosystems) and first-strand cDNA synthesis was performed using oligo(dT)s and Maxima H Minus reverse transcriptase (Thermo Scientific) per the manufacturer's instructions. Quantitative real-time PCR was performed using SsoFast EvaGreen Supermix (Bio-Rad) and the Bio-Rad CFX96 Real-Time detection system per manufacturer's instructions. Each sample was normalized to GAPDH (ΔCT) and expressed as a fold change relative to controls via the ΔΔCT method. Primers were purchased from Integrated DNA Technologies and the sequences were as follows: 5′–GACATCAAGAAGGTGGTGAAGC-3′ and 5′–TCCAGGGTTTCTTACTCCTTGG-3′ for GAPDH and 5′–GGAGTTGAGAATGCTGAGAG–3′ and 5′–TCCAGGGTTTCTTACTCCTTGG–3′ for VP1.

### Western blotting analysis

Cells and islets were washed with PBS and lysed in Laemmli buffer, proteins were separated by SDS-PAGE and Western blotting analysis was conducted as previously described (68). Primary and secondary antibodies were used at the following dilutions: mouse anti-GAPDH, 1:10,000; mouse anti-3D^pol^ 1:1000; rabbit anti-p-AMPK (Thr-172), 1:1000; donkey anti-mouse antibody-horseradish peroxidase, 1:20,000; donkey anti-rabbit antibody-horseradish peroxidase, 1:20,000. Bands were detected using chemiluminescence.

### Nucleotide measurement

Nucleotides (ATP) were extracted using perchloric acid precipitation and quantified using HPLC (HPLC) analysis as previously described (69, 70). Protein concentration was determined using the Thermo Scientific Pierce BCA Protein Assay Kit. Nucleotide levels were normalized to total protein and expressed as a percent relative to the untreated control.

### Immunofluorescence

Dispersed islet cells were washed twice in PBS, resuspended in PBS, and centrifuged onto microscope slides using a Shandon Cytospin II (ThermoFisher Scientific). Cells were fixed with 4% paraformaldehyde for 15 min, permeabilized with 0.2% Triton X-100 in PBS for 30 min and blocked using 1% BSA in PBS with 0.2% Tween (PBST). Primary antibodies to insulin and EMCV capsid were used at 1:1000 in PBST for 1 h. Secondary antibodies Cy3-conjugated donkey anti-guinea pig and AF-488-conjugated donkey anti-rabbit were used at 1:1000 in PBST in a dark, humidified chamber for 1 h. ProLong^TM^ Gold Antifade Reagent with DAPI (Invitrogen) was used to preserve fluorescent signal and for nuclear staining. Images were captured using a Nikon eclipse 90i confocal microscope.

### Statistics

Statistical comparisons were made between groups using either one- or two-way analysis of variance (ANOVA). Significant differences between groups were determined using the Tukey-Kramer post-hoc test or Dunnett's multiple comparisons test. Statistically significant differences (*p* < 0.05) are indicated, *.

## Data Availability

All the data are contained in the manuscript.
